# Impact of airflow limitation in chronic heart failure

**DOI:** 10.1007/s12471-017-0965-4

**Published:** 2017-02-27

**Authors:** S. Bektas, F. M. E. Franssen, V. van Empel, N. Uszko-Lencer, J. Boyne, C. Knackstedt, H. P. Brunner-La Rocca

**Affiliations:** 1 Department of Cardiology, Maastricht University Medical Center, Maastricht, The Netherlands; 2 Department of Respiratory Medicine, Maastricht University Medical Center, Maastricht, The Netherlands; 3 Department of Research and Education, CIRO+, Center of expertise for chronic organ failure, Horn, The Netherlands

**Keywords:** Prevalence, Chronic obstructive pulmonary disease, Chronic heart failure, Diagnosis, Quality of life

## Abstract

**Background:**

Comorbidities are common in chronic heart failure (HF) patients, but diagnoses are often not based on objective testing. Chronic obstructive pulmonary disease (COPD) is an important comorbidity and often neglected because of shared symptoms and risk factors. Precise prevalence and consequences are not well known. Therefore, we investigated prevalence, pulmonary treatment, symptoms and quality of life (QOL) of COPD in patients with chronic HF.

**Methods:**

205 patients with stable HF for at least 1 month, aged above 50 years, were included from our outpatient cardiology clinic, irrespective of left ventricular ejection fraction. Patients performed post-bronchodilator spirometry, a six-minute walk test (6-MWT) and completed the Kansas City Cardiomyopathy Questionnaire (KCCQ). COPD was diagnosed according to GOLD criteria. Restrictive lung function was defined as FEV_1_/FVC ≥0.70 and FVC <80% of predicted value. The BODE and ADO index, risk scores in COPD patients, were calculated.

**Results:**

Almost 40% fulfilled the criteria of COPD and 7% had restrictive lung disease, the latter being excluded from further analysis. Noteworthy, 63% of the COPD patients were undiagnosed and 8% of those without COPD used inhalation therapy. Patients with COPD had more shortness of breath despite little difference in HF severity and similar other comorbidities. KCCQ was significantly worse in COPD patients. The ADO and BODE indices were significantly different.

**Conclusion:**

COPD is very common in unselected HF patients. It was often not diagnosed and many patients received treatment without being diagnosed with COPD. Presence of COPD worsens symptoms and negatively effects cardiac specific QOL.

## Introduction

Heart failure (HF) and chronic obstructive pulmonary disease (COPD) are amongst the leading causes of morbidity and mortality in Western countries. Until recently, COPD and HF have mostly been studied individually, but during the last years, the awareness of the interaction of both diseases has grown. Studies showed that HF and COPD frequently coincide, but precise epidemiologic data are lacking. In fact, the reported prevalence of COPD in HF patients varied substantially between 9–52% [1–4]. In part, this may be caused by the lack of systematic lung function testing.

Diagnosing COPD is challenging, especially in patients with HF. Not only do the two diseases share major symptoms such as dyspnoea and fatigue, they also have common etiological factors such as smoking and ageing [5–8]. Consequently, under- and over-diagnosing COPD in HF patients are common [9–12]. Also from a therapeutic and prognostic perspective, the coincidence of COPD and HF is relevant. Many patients with HF are treated with inhalation therapy, although no proper diagnosis of COPD has been made [12]. Accordingly, patients may be treated inadequately, but the extent of this has not yet been properly addressed. Since both diseases result in significantly reduced quality of life and mortality, proper diagnosis, and consequently treatment, may result in considerably improved well-being of these patients [13–15].

Therefore, this study aimed to address those clinically important shortcomings by investigating consecutive patients diagnosed with and treated for chronic HF, irrespective of left ventricular ejection fraction (LVEF), with respect to the prevalence of COPD, treatment and the clinical consequences thereof. Moreover, we aimed to identify patients at risk for airflow limitation compatible with COPD.

## Methods

### Study design and participants

Consecutive patients visiting the outpatient HF clinic of the Maastricht University Medical Centre (the Netherlands) were screened for inclusion in this cross-sectional observational study between October 2012 and November 2013 and were asked to participate in this study. Inclusion criteria were documented HF based on prevailing European Society of Cardiology guidelines [16] with left ventricular dysfunction (LVD), irrespective of LVEF, age above 50 years and clinically stable condition for at least one month. Patients who were not able to cooperate or in whom spirometry was clinically contraindicated (detached retina, active tuberculosis, resting pulse >120/min) were excluded. Other exclusion criteria were recent surgery, recent myocardial infarction (<1 month), lower respiratory tract infection or pneumothorax within the last 2 months or stroke within the last 12 months. The study was approved by the local ethics committee. All patients provided written informed consent prior to enrolment.

### Measurements and data collection

#### Clinical characteristics

The following clinical characteristics and symptoms were extracted from the patient charts: age, gender, cause of HF, other cardiovascular diseases, co-morbidities, cardiovascular risk factors (diabetes, hypertension, hypercholesterolaemia, smoking), healthcare utilisation in the past year, NYHA class, weight and height, hip/waist circumference, blood pressure, heart rate, ECG (rhythm, QRS complex duration), currently used medication, in particular cardiac and pulmonary medication in detail, recent antibiotic and glucocorticosteroid use for respiratory symptoms/infections. Also, (non-invasive) O_2_-saturation at rest was measured. Two years after the inclusion of the first patient, medical charts were retrospectively reviewed to determine all-cause mortality.

#### Pulmonary assessment

Lung function was measured in all patients after 200 μg of salbutamol was given. Spirometry (Masterscreen, Jaeger, Würzburg, Germany) was performed by fully trained respiratory technicians according to ERS standards for acceptability and reproducibility [17]. Airflow limitation, compatible with COPD, was defined according to the Global initiative for Obstructive Lung Disease (GOLD) report [18] as a ratio between forced exhaled volume in the first second (FEV_1_) and forced vital capacity (FVC) less than 0.70 after bronchodilatation. According to GOLD, the severity of airflow limitation was staged as mild, moderate, severe and very severe if percent predicted FEV_1_ was >80%, 50–80%, 30–50%, or <30%, respectively [18]. Patients fulfilling the diagnostic criteria for COPD, were subsequently stratified according to symptoms and future risk [18]. For this classification, COPD Assessment Test (CAT) scores were used as the preferred symptom measure [19]. Restrictive lung disease was defined as post-bronchodilator FEV_1_/FVC ≥0.70 and FVC <80% of predicted, while normal lung function was defined as neither airflow limitation nor restrictive lung disease. A six-minute walk test (6-MWT) was carried out according to international standards [20].

#### Cardiac evaluation

We collected the results of comprehensive standardised echocardiographic (Philips IE33) examination performed within 6 months prior to study enrolment. If no recent echocardiography was available, it was repeated for this study. Assessment included left ventricular end-systolic and end-diastolic dimensions and function, dimensions of atria, right ventricle dimension and function, valvular functions, and estimate of systolic pulmonary arterial pressure (gradient across tricuspid valve, as well as dimension and variation of vena cava inferior). HF was split into two groups, based on the LVEF. Thus, HF with preserved LVEF (HFpEF) has been defined as the presence of typical HF signs and symptoms, evidence of diastolic dysfunction (abnormal LV relaxation or diastolic stiffness) and presence of normal or only mildly abnormal LV systolic function, i. e., LVEF of more than 50%. Patients with HF signs and symptoms and LVEF of <50% were defined as having HF with reduced LVEF (HFrEF) [16].

#### Questionnaires

Patients completed the Kansas City Cardiomyopathy Questionnaire (KCCQ) [21], a 23-item questionnaire that quantifies physical limitations, symptoms, self-efficacy, social interference and quality of life. Dyspnoea was quantified by the modified Medical Research Council (mMRC) scale [22]. Health status was assessed with the CAT, which is a COPD-specific health status questionnaire [19]. In addition, Body mass index, airflow Obstruction, Dyspnoea, and Exercise capacity (BODE) [23] and Age, Dyspnoea, airflow Obstruction (ADO) [24] indices were calculated. Where the distance walked in six minutes (6-MWT) was not known, 350 m was used to calculate the score. Where mMRC was missing, NYHA class was used.

### Statistical analysis

Descriptive data are presented as mean (±standard deviation – SD), frequencies (%) or median (interquartile range – IQR), as appropriate. Baseline characteristics of patients with and without COPD were compared using independent t‑test or Mann-Whitney U test for continuous variables and Chi-square or Fisher’s exact test for categorical variables, as appropriate. Survival analyses were done using Kaplan-Meier curves and groups were compared using the log-rank test. A two-sided *p*-value of less than 0.05 was considered as statistically significant. We used the commercially available statistical package SPSS v 22.0 (IBM) for analyses.

## Results

### Patient characteristics

**Fig. 1:**
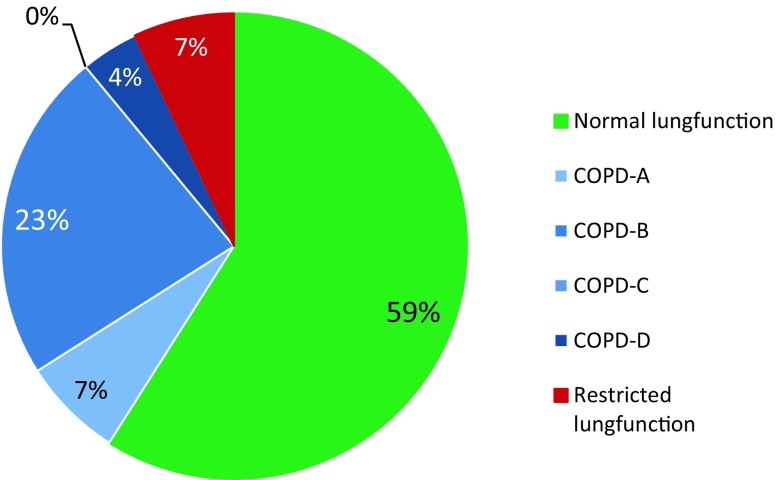
Distribution of new GOLD classification A–D in heart failure patients. (*COPD* chronic obstructive pulmonary disease, *GOLD* Global initiative for chronic Obstructive Lung Disease)

A total of 586 patients were screened. Of these, 205 HF patients volunteered to participate in this study. Six patients did not complete a lung function test and were excluded for further analysis. Of the remaining 199 patients, 13 patients (7%) had restrictive lung disease and were not analysed further (Fig. 1).

The demographic and clinical characteristics are shown in Table 1. The patients had an overall mean age of 76 years, median LVEF of 44% and approximately 70% of the participants were male. The mean ejection fraction for HFrEF patients was 37%. Thirty percent of the patients had HFpEF. Approximately half of the patients had mild symptoms (NYHA class II) and 50% had an ischaemic aetiology of HF. Other most common causes of HF were dilated cardiomyopathy (19%) and hypertensive heart disease (18%). Most patients were former or non-smokers.

**Table 1:** Baseline characteristics of the heart failure patients with or without COPD

	All (*n* = 186)	No COPD (*n* = 118)	COPD (*n* = 68)	*P*-value
Age, years	76 [68–82]	76 [67–82]	76 [69–84]	0.55
Male sex	129 (69%)	78 (66%)	51 (75%)	0.25
BMI, kg/m^2^	29 (±5)	29 (±5)	28 (±5)	0.08
LVEF, % Preserved EF	44 [32–54] 57 (31%)	46 [32–57] 40 (38%)	43 [30–50] 17 (27%)	0.11 0.18
*NYHA class*				**0.014**
NYHA I NYHA II NYHA III NYHA IV	47 (25%) 99 (53%) 38 (20%) 2 (1%)	38 (32%) 61 (52%) 18 (15%) 1 (1%)	9 (13%) 38 (56%) 20 (29%) 1 (2%)	
*Cause of HF*				0.33
CAD DCM HHD	91 (49%) 35 (19%) 33 (18%)	51 (43%) 25 (21%) 23 (20%)	40 (59%) 10 (15%) 10 (15%)	
*Co-morbidity*				
Hypertension Diabetes mellitus Hyper-Cholesterolaemia CVA/TIA PAD	114 (61%) 49 (26%) 133 (72%) 17 (9%) 6 (3%)	77 (65%) 32 (27%) 86 (73%) 11 (9%) 3 (3%)	37 (54%) 17 (25%) 47 (69%) 6 (9%) 3 (4%)	0.16 0.86 0.62 1.0 0.67
*Smoking history*				**0.001**
Non-smoker Current smoker Former smoker	49 (26%) 22 (12%) 106 (57%)	42 (37%) 10 (9%) 61 (54%)	7 (11%) 12 (19%) 45 (70%)	
Symptoms and clinical findings *Blood pressure*				
– Systolic	125 (±21)	129 (±21)	118 (±19)	**<0.001**
– Diastolic	69 (±11)	70 (±11)	68 (±11)	0.29
– Pulse	71 [62–80]	70 [62–80]	72 [64–81]	0.55
O_2_ saturation (*n* = 134)	97 [96–98]	98 [97–98]	97 [96–98]	**0.005**
NT-proBNP	93 [33–229]	81 [28–205]	98 [42–247]	0.50
History of COPD I–IV	28 (15%)	3 (3%)	25 (37%)	**<0.001**
History of asthma	7 (4%)	2 (2%)	5 (7%)	0.10
Exacerbation last year	6 (3%)	1 (1%)	5 (7%)	**0.02**
Inhalation therapy without proper diagnosis	14 (8%)	7 (6%)	7 (10%)	0.39
Inhalation therapy	34 (18%)	10 (9%)	24 (35%)	**<0.001**
*Cardiac medication*				
ACE-I ARB β-blockers Diuretics Aldosterone antagonists	110 (59%) 59 (32%) 172 (93%) 147 (79%) 74 (40%)	70 (59%) 35 (30%) 109 (92%) 91 (77%) 42 (36%)	40 (60%) 24 (35%) 63 (93%) 56 (82%) 32 (47%)	1.0 0.51 1.0 0.46 0.16
*Spirometry*				
FEV_1_, L FEV_1_, % predicted FVC, L FVC, % predicted FEV_1_/FVC, %	2.4 (±0.8) 94 (±23) 3.4 (±0.90) 103.7 (±17) 71 (±12)	2.7 (±0.7) 105 (±17) 3.4 (±0.9) 106.2 (±15) 78 (±5)	2.0 (±0.64) 75 (±21) 3.4 (±0.9) 99.5 (±20) 58 (±9)	**<0.001** **<0.001** 0.97 **0.01** **<0.001**

Data are presented as number (%), mean (±SD) and median [IQR], unless specified otherwise. *P*-values below 0.05 in bold

*BMI* body mass index, *CAD* coronary artery disease, *DCM* dilated cardiomyopathy, *HHD* hypertensive heart disease, *NYHA* New York Heart Association class, *CVA* cerebrovascular accident, *TIA* transient ischemic attack, *PAD* peripheral arterial disease, *NT-proBNP N*-terminal pro-brain natriuretic peptide, *COPD* chronic obstructive pulmonary disease, *FVC* forced vital capacity, *FEV*_1_ Forced expiratory volume in 1 s, *ACE-I* angiotensin converting enzyme inhibitor, *ARB* angiotensin receptor blocker, *β-blockers* beta-adrenergic blocking agents

### COPD prevalence

Of the 186 patients without restrictive lung disease, 68 (37%) fulfilled the criteria of COPD. According to the traditional GOLD classification, 29 (15%) patients had mild, 31 (16%) moderate and 8 (4%) severe disease, while no patients had very severe COPD. When the updated GOLD assessment was applied, most COPD patients were categorised as GOLD B (Fig. 1). The prevalence of COPD did not differ between men and women.

Importantly, 43 (63%) patients were previously undiagnosed with COPD, whereas 3 of 118 patients (2.5%) who did not have significant airflow limitation had received the diagnosis of COPD previously. In addition, 8% of the patients used pulmonary medication without a proper diagnosis of COPD.

Patients with COPD had more symptoms of dyspnoea as compared to those having HF only. Systolic blood pressure and saturation differed significantly. The number of co-morbidities was similar in both groups. The proportion of patients with a previous diagnosis of asthma was not significantly different between HF patients with and without COPD. The distance walked during the 6‑MWT did not differ significantly (Table 2).

**Table 2:** Dyspnoea assessment and 6‑minute walk test in heart failure patients with or without COPD

	All (*n* = 186)	No COPD (*n* = 118)	COPD (*n* = 68)	*P*-value
*mMRC scale*				**0.006**
0 1 2 3 4	29 (16%) 74 (40%) 31 (17%) 42 (23%) 10 (5%)	23 (20%) 52 (44%) 17 (14%) 24 (20%) 2 (2%)	6 (9%) 22 (32%) 14 (21%) 18 (27%) 8 (12%)	
CAT score (*n* = 156)	14 [9–20]	13 [8–19]	16 [11–22]	0.07
6-MWT, m (*n* = 141)	397 [318–460]	402 [308–462]	393 [318–442]	0.68

Data are presented as number and median [IQR], unless specified otherwise. *P*-values below 0.05 in bold

*CAT* COPD assessment test, *6-MWT* six-minute walk test, *mMRC* modified Medical Research Council scale

The medication used in both groups was similar, except for the inhalation therapy at baseline, which was more frequently used in the COPD patients (Table 1).

### Quality of life and survival

The difference in the CAT score between HF patients with or without COPD failed to reach statistical significance (Table 2). The overall KCCQ scores were significantly worse in the COPD group (*p* = 0.005) as summarised in Table 3. The following domains were significantly worse in the COPD group: physical limitation, symptom severity and symptom stability. The other domains did not significantly differ between patients with and without COPD. The ADO and BODE index were significantly different between the two groups (Table 3).

**Table 3:** Quality of life in heart failure patients with or without COPD

QOL	Overall (*n* = 186)	No COPD (*n* = 118)	COPD (*n* = 68)	*P*-value
KCCQ score	78 (±20)	82 (±19)	73 (±21)	**0.005**
Physical limitation	52 (±16)	54 (±15)	47 (±16)	**0.002**
Symptoms	75 (±24)	79 (±24)	67 (±22)	**0.001**
Symptom stability	44 (±13)	46 (±13)	41 (±13)	**0.022**
Social limitation	62 (±21)	64 (±21)	58 (±20)	0.08
Self-efficacy	78 (±18)	80 (±17)	74 (±21)	0.11
Quality of life	65 (±19)	67 (±19)	61 (±19)	0.08
ADO score	4[3–5]	4[3–4]	4[3–6]	**0.001**
BODE score	1[0–4]	0[0–3]	2[0–5]	**0.004**

Mean (±SD), median [IQR]. *P*-values below 0.05 in bold

*COPD* chronic obstructive pulmonary disease, *ADO* Age, Dyspnoea and airflow Obstruction, *BODE* Body-mass index, airflow Obstruction, Dyspnoea, and Exercise capacity, *QOL* quality of life, *KCCQ* Kansas City Cardiomyopathy Questionnaire

**Fig. 2:**
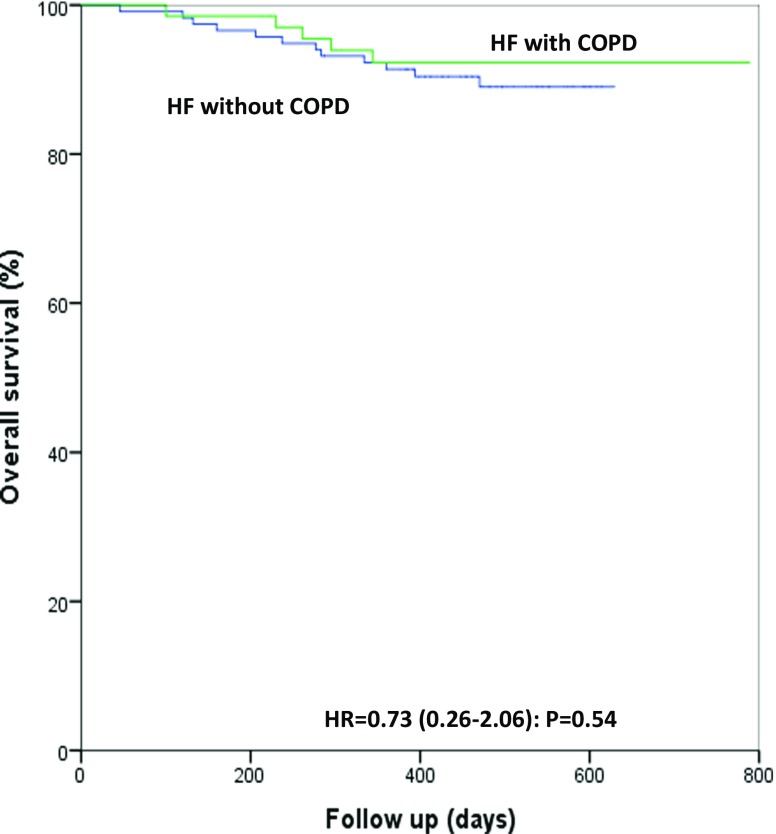
Kaplan-Meier survival plot in heart failure patients with or without COPD. (*COPD* chronic obstructive pulmonary disease, *GOLD* Global initiative for chronic Obstructive Lung Disease, *HF* heart failure, *HR* hazard ratio (95% confidence interval), *p*-value derived from log-rank test)

Survival did not differ significantly between HF patients with COPD and without COPD (*p* = 0.54) (Fig. 2).

## Discussion

This study showed a high prevalence of COPD in HF patients, which was comparable for HFrEF and HFpEF. Noteworthy, HF patients with concomitant COPD experienced significantly more symptoms of dyspnoea and had worse quality of life compared to those without COPD, despite no evidence of differences in severity of HF. This may be of particular clinical importance as underdetection of COPD was common. On the other hand, some patients were treated with pulmonary medication without a proper diagnosis.

### Prevalence of COPD in heart failure

In previous studies, the prevalence of COPD varied widely from 9 to 56% in patients with chronic HF, being on average between 20 and 30% [12, 25–27]. Most studies were retrospective and included decompensated or hospitalised HF patients [8, 11, 12, 25–27]. Studies in stable chronic HF patients are, however, relatively scarce. Valk et al. [12], reported COPD prevalence of 28% in 106 primary care patients. Boschetto et al. [26] reported a prevalence of 30% in 118 predominantly male, ambulatory HF patients. Masceranhas et al. [27] included 186 HFrEF patients retrospectively and reported a COPD prevalence rate of 40%. The variation in prevalence rates may depend on factors such as population, study design, inclusion criteria and used diagnostics [28]. In particular, most studies did not systematically perform lung function testing and based the diagnosis on medical history alone. In the current study, state of the art spirometry was performed in all HF patients. Given the significant proportion of underdiagnosis of COPD, this very likely explains the somewhat higher prevalence in this study. Moreover, the prevalence of COPD in HF patients increases with age until approximately 75 years [28, 29]. Thus, the mean age of >75 years in this study may have contributed to the observed prevalence.

HF itself can cause a reduction of about 20% in FEV1 and FVC [30]. Therefore, some studies recommend the use of the individual lower limit of normal (LLN), instead of the GOLD classification, to define COPD to prevent overdiagnosis of COPD in the elderly HF patients [31–33]. However, in stable, not volume overloaded patients, the ratio FEV1/FVC is almost similarly impaired, thus the ratio FEV1/FVC is not affected prominently when spirometry is performed in a stable condition of the disease [30, 33]. This study included only stable HF patients; overdiagnosing COPD by using the fixed FEV1/FVC ratio proposed by GOLD in this study is therefore very unlikely [30, 34, 35]. Furthermore, the current guidelines acknowledge the limitations of the current GOLD-COPD classification, but appropriate alternatives are lacking [18].

Prevalence of COPD in HF patients with HFpEF was reported to be higher in comparison to HF with HFrEF [28]. This study did not confirm such a difference between patients with HFpEF and HFrEF. In fact, patients with preserved ejection fraction even tended to have less concomitant COPD. There are several reasons that could explain this discrepancy. Thus, most of the previous reports were retrospective, did not use proper testing, were predominantly performed in hospitalised patients and cut-off regarding ejection fraction was not uniform [36–42]. Moreover, inclusion of HFpEF patients who actually have HF is certainly challenging and misdiagnosis exists [43, 44]. Thus, reliable estimates of COPD prevalence in representative stable HFpEF patients are still lacking [28]. Therefore, this study gives important insight in the COPD prevalence in the HFpEF population in comparison to other studies. Given the results of this study, showing that diagnosis of COPD is often missed or performed without proper testing, and the same is true for HFpEF, it may be speculated that some COPD patients may be misdiagnosed as HFpEF (and vice versa) in previous cohorts, highlighting the need for proper diagnostics, particularly in patients with suspicion of HFpEF.

### Impact on symptoms and quality of life

The presence of COPD appears not only to worsen dyspnoea, but also negatively affect cardiac specific quality of life, as seen by the results of the KCCQ. On the other hand, the presence of COPD in HF patients did not negatively affect pulmonary specific health status, as assessed by the CAT questionnaire. Still, there was a strong trend which just failed to reach statistical significance, possibly due to less power as a result of missing values. Unfortunately, there is no specific questionnaire for patients with HF and concomitant COPD. In this regard, there is room to investigate and compare which questionnaire is suited best for such patients, or to develop a new questionnaire that specifically addressing symptoms and quality of life for patients suffering from both HF and COPD.

Despite having more symptoms, the functional status was not different between the two groups as assessed by the 6MWT [20]. This contrast with a previous study and the discrepancy cannot easily be explained. The average distance walked in this study indicates mild to moderate limitation in functional capacity, probably primarily due to HF.

### Impact on prognosis

Even though the effect on survival is negligible, current treatment of COPD generates symptom relief and improves quality of life [45, 46]. Therefore, it may be expected that proper treatment of COPD may result in improvement of symptoms and quality of life. Still, it needs to be prospectively tested if this really is the case. On the other hand, up to now it is unknown which specific COPD treatment is the best in HF patients. Advantages of HF medication in comorbid COPD patients on survival are well established, but the other way around is not that well established. Caution is needed with some inhalation therapy in HF patients [47–49]. Therefore, safety and efficacy of various COPD drugs needs to be tested in appropriate trials. In addition, cessation of unnecessary treatment in patients not having COPD is of utmost importance. In this study, some patients received pulmonary medication without a proper diagnosis. Finally, survival was not adversely affected by COPD in this study. However, the study was too small to address this question sufficiently. Previous reports suggested some impact, but results were not uniform. Again, inhomogeneous criteria for diagnosis and differences in treatment may explain such discrepancies, further stressing the need of prospective testing of best management of these patients in a large prospective trial.

## Strengths and limitations

A strength of this study is the inclusion of a representative group of stable chronic HF patients with both preserved and reduced ejection fraction, independent of their smoking status. Importantly, all patients underwent both lung function testing and had echocardiography. Therefore, diagnosis of both COPD and HF was accurate. A limitation is the relatively limited sample size. Moreover, some exclusion criteria applied and not all patients gave informed consent for participation. Therefore, we can exclude some selection bias. The relatively good prognosis in our cohort shows that we included stable HF patients and results might be different in less stable patients or patients with more severe HF.

## Conclusion

In conclusion, this study demonstrated that 40% of HF patients may have concomitant pulmonary disease, predominantly mild to severe COPD. Importantly, these patients had more severe symptoms of dyspnoea and worse quality of life than those without COPD. Many patients with COPD were undetected and some were treated for pulmonary disease without proper diagnosis, or received the wrong treatment. Thus, the findings of this study underline the importance of systematically identifying patients with COPD who are diagnosed with HF.
